# Consistency Between Clinical Trial Registry Entries and Journal Publications in Transfusion Medicine: An Observational Study

**DOI:** 10.3390/jcm15103981

**Published:** 2026-05-21

**Authors:** Iva Jerčić Martinić-Cezar, Shelly Melissa Pranić, Ante Tavra, Ana Marušić

**Affiliations:** 1 Center for Transfusion Medicine, University Hospital of Split, Spinčićeva 1, 21000 Split, Croatia; 2 Department of Public Health, University Hospital of Split, Spinčićeva 1, 21000 Split, Croatia; shelly.pranic@mefst.hr; 3 Department of Pediatrics, Section of Medical Genetics, University Hospital of Split, Spinčićeva 1, 21000 Split, Croatia; ante.tavra@kbsplit.hr; 4 Center for Evidence-Based Medicine, Department of Research in Biomedicine and Health, University Hospital of Split, Spinčićeva 1, 21000 Split, Croatia; ana.marusic@mefst.hr; 5 Department of Science, University Hospital of Split, Spinčićeva 1, 21000 Split, Croatia

**Keywords:** transfusion medicine, clinical trial registration, *ClinicalTrials.gov*, WHO trial registration data set, reporting consistency, adverse event reporting

## Abstract

**Background/Objectives**: Transparent and complete reporting of clinical trial information across registries and peer-reviewed publications is essential for reliable interpretation of clinical evidence. Previous studies have demonstrated discrepancies between trial registries and journal publications, but data specifically focusing on transfusion medicine trials remain limited. To assess reporting completeness and consistency for key WHO Trial Registration Data Set (WHO TRDS) items and safety outcomes across the trial life cycle in transfusion medicine-related clinical trials. **Methods**: We conducted a retrospective observational study of completed transfusion medicine-related clinical trials registered in ClinicalTrials.gov, with registry results available between January 2009 and May 2019. Reporting of WHO TRDS items was evaluated at three predefined time points: initial registry entry, final registry update, and corresponding peer-reviewed journal publication. Changes and missing items were systematically assessed, and adverse event and mortality reporting were compared between registry records and journal publications. **Results**: A total of 67 eligible clinical trials were identified, of which 45 (67%) had corresponding peer-reviewed journal publications. At initial registration, several WHO TRDS items were frequently missing, particularly timeline- and outcome-related fields. Completeness improved substantially in final registry updates but remained inconsistent in journal publications, where discrepancies in eligibility criteria, outcome definitions, and study timelines were common. Differences between final registry updates and publications were observed in the majority of trials. Safety reporting also differed between sources: serious adverse events were reported in 31/45 (69%) registry entries and 26/45 (58%) publications, whereas deaths were more frequently reported in publications (27/45, 60%) than in registries (20/45, 44%). **Conclusions**: Clinical trials in transfusion medicine show inconsistencies between registry records and corresponding journal publications across key methodological and safety reporting domains. These differences may limit transparency, reproducibility, and the reliability of evidence synthesis. Closer alignment between trial registries and scientific publications is needed to strengthen the trustworthiness of clinical information in transfusion medicine.

## 1. Introduction

Transfusion therapy is an integral part of daily medical practice [1], involving the use of blood products and representing one of the most frequently performed procedures in healthcare [2], as well as the evolving application of non-transfusion therapies [3]. Although blood transfusions are safer than ever and provide substantial clinical benefits, with most procedures occurring without complications [4], certain risks remain despite continuous advancements across all areas of transfusion medicine. In addition, emerging challenges continue to pose potential threats to transfusion safety [5].

High-quality clinical research plays an important role in guiding safe and effective transfusion practice [6]. Clinical trials, particularly well-designed prospective randomized studies, provide the methodological foundation needed to evaluate the safety and efficacy of blood components [7] and to support the appropriate use of cellular therapies, hemostatic agents, and other pharmacological interventions used in transfusion medicine [8]. However, the current literature indicates that the clinical evidence in transfusion medicine is still not sufficiently developed, leaving several important areas underinvestigated [9]. This is noteworthy because clinical trials remain the preferred design for assessing therapeutic and preventive interventions [10]. Their value depends on clear, transparent, and complete reporting, which is necessary for accurate clinical interpretation and reliable evidence synthesis [11,12]. Persistent deficiencies in reporting both planned and completed clinical trials continue to weaken the trustworthiness and credibility of non-transparent publications [13,14]. This problem has been described across multiple medical disciplines, including transfusion medicine [15,16,17,18,19].

Selective outcome reporting in clinical trials has generated concern regarding the credibility of data presented in peer-reviewed journal publications as a basis for clinical decision-making [20]. Trial registration has been introduced as a key strategy to reduce dissemination and reporting biases [21]. Since September 2009, the reporting of adverse events (AEs) in *ClinicalTrials.gov* has become a legal requirement in the USA [22], as stipulated by Section 801 of the Food and Drug Administration Amendments Act (FDAAA 801) of 2007 and implemented through the Final Rule in 2017 [23]. Under this regulation, clinical trial sponsors and investigators are required to provide tabular summaries of expected and unexpected serious adverse events (SAEs), other adverse events (OAEs), and data on all-cause mortality (ACM) [24]. These summaries are intended to correspond to AE data reported in journal articles describing clinical trials. However, evidence from multiple medical fields indicates that journal publications often underreport or inconsistently report AEs, including clinically important safety outcomes and adverse drug reactions, in contrast to the Consolidated Standards of Reporting Trials (CONSORT) guidelines [25,26,27,28,29,30].

Despite these regulatory and methodological developments, data evaluating the reliability of reporting in clinical trials related to transfusion medicine remain limited [31]. The aim of this study was to evaluate the completeness and consistency of reporting of selected WHO Trial Registration Data Set items and safety outcomes across the trial life cycle in transfusion medicine-related clinical trials, including comparisons between initial registry entries, final registry updates, and corresponding peer-reviewed journal publications.

## 2. Methods

### 2.1. Study Periods and Data Sources

We conducted a retrospective analysis of completed transfusion medicine-related clinical trials registered in ClinicalTrials.gov, with registry results available between 1 January 2009 and 31 May 2019. The search for corresponding publications was performed repeatedly, with the final update conducted in November 2025. During data extraction and verification, *ClinicalTrials.gov* registry records were re-evaluated in 2025 using the archive/version history feature (“Study Record Versions”). Initial registration records and final registration updates were reviewed for each included study. Comparisons with corresponding publications were performed using the final registration update available at the time of reassessment. *ClinicalTrials.gov* was selected as the largest registry, providing well-structured and curated information across all stages of trial registration, as well as clear legal requirements for safety data reporting [32]. The study adhered to the Strengthening the Reporting of Observational Studies in Epidemiology (STROBE) guidelines [33].

### 2.2. Sample

Requirements for considering trials as applicable clinical trials according to the FDAAA 801 were verified according to “Elaboration of definitions of responsible party and applicable clinical trial (ACT)” from 9 March 2009 for trials initiated after 27 September 2007 and according to “Checklist for evaluating whether a clinical trial or study is an ACT” for those initiated after 18 January 2017 [34]. Responsible parties conducting ACT are legally mandated to report their results, including all adverse events, to *ClinicalTrials.gov* within 12 months of the trial’s primary completion date, regardless of whether the findings have been published in a journal. This requirement is stipulated by the FDA Amendments Act of 2007 (FDAAA 801) and reinforced by the Final Rule effective from January 2017 to include mortality reporting [34].

We searched the registry for completed clinical trials using the keyword “transfusion” to capture a subset of transfusion-related trials rather than a comprehensive or globally representative sample. We did not use Medical Subject Headings (MeSH) terms. Additional inclusion criteria were applied by selecting specific filters in the registry: (1) interventional studies, to focus on trials testing specific interventions, (2) trials with available registry results, since this criterion was essential for comparing registry data with corresponding publications, and (3) trials marked as “Completed” to ensure that the results and safety data had been fully collected and reported. Trials that did not meet the inclusion criteria were excluded: trials without available registry results, trials with unknown status, trials that were still recruiting, trials not actually investigating transfusion medicine, and trials classified as Phase 1 or “Phase N/A” during manual eligibility verification of registry records. Phase 1 and Phase N/A studies were excluded to maintain a more methodologically consistent cohort of phase-based therapeutic trials for comparative analyses of reporting characteristics and adverse event data across registry entries and corresponding publications. Phase N/A studies primarily included device-, procedure-, or blood component-processing-related interventions, which may differ methodologically from conventional phase-based therapeutic trials.

For trials with multiple publications listed on *ClinicalTrials.gov*, we included only the first full publication that reported the main trial results related to the primary outcome. This approach was intended to capture the initial and primary reporting of adverse events for each trial. We excluded secondary publications, focusing exclusively on the primary report to evaluate the consistency and completeness of safety reporting between *ClinicalTrials.gov* and the corresponding journal publications. Corresponding publications were first identified by screening citations provided in *ClinicalTrials.gov*. The Publication(s) subheading under the Descriptive information heading of the Tabular View in *ClinicalTrials.gov* was reviewed to identify only articles discussing the results of the clinical trial on transfusion medicine, disregarding all those which provided only related background information. When a publication was not listed in the trial registry, we searched for it using a two-step search strategy in databases including PubMed, Web of Science, Scopus, and Google Scholar. Firstly, we used the [si] tag along with the NCT number [35] which is typically found in the abstract or body of published articles. If no results were found through this method, we expanded our search using the principal investigator’s name, study title, study duration, and the trial’s country of origin. Comparisons between registry data and publications were based on full articles, including any Supplementary Materials. We considered a publication to correspond to the registered trial if five of the following six criteria matched: study design, drug interventions, primary outcomes, condition, enrolment and study location. Only full publications were compared with registered data. In cases where correspondence between registry records and publications remained uncertain, records were reviewed jointly by the investigators and resolved through consensus discussion.

### 2.3. Data Extraction and Comparisons

We extracted the data related to the completeness of trial registration based on selected data items from the World Health Organization Trial Registration Data Set (WHO TRDS), version 1.3.1 [36]. WHO TRDS v1.3.1 was used as a structured descriptive framework for evaluating reporting completeness and consistency across registry entries and publications rather than as a formal compliance benchmark for all trials included in the cohort.

Of the 24 TRDS items, 15 were evaluated in this study: trial identifying number, primary sponsor, public title, scientific title, countries of recruitment, health condition studied, interventions, key inclusion and exclusion criteria, study type, date of first enrolment, sample size, key primary outcomes, key secondary outcomes, and completion date. The IPD sharing statement was evaluated where applicable, according to the implementation of this requirement in trial registration records and publications. Because all trials were sourced from *ClinicalTrials.gov*, item 1 was operationalized as the NCT identifier. Although automatically assigned by the registry, it was included in the analysis to assess whether the registration number was consistently reported in corresponding publications.

TRDS items 2, 3, 4, 6, 7, 8, 18, and 21 were not evaluated because they primarily represent administrative information, may change over time, are automatically generated at registration, or cannot be consistently assessed across registry records and corresponding publications.

In accordance with the WHO TRDS framework, key inclusion and exclusion criteria were considered a single TRDS item; however, they were extracted and presented separately where appropriate to enable a more detailed assessment of missing information and discrepancies between registry records and publications. Discrepancies were assessed across predefined WHO TRDS items and categorized as changed, added, or deleted. These categories captured substantively relevant differences, including modifications of reported information (changed) and the presence or absence of information between registry entries and corresponding publications (added or deleted). Minor differences in wording that did not alter meaning (e.g., abbreviations or stylistic variations) were not classified as discrepancies. The classification framework was intended to identify differences between registry entries and publications but was not designed to distinguish between differences arising from legitimate protocol amendments, registry updating practices, editorial modifications, or potentially selective reporting.

Adverse event reporting, corresponding to the WHO TRDS Summary Results item in *ClinicalTrials.gov*, was analyzed separately from the WHO TRDS registration items and compared with reporting in the corresponding peer-reviewed journal publications.

Secondary outcomes included:

(1) changes in the reporting of WHO TRDS items between initial and final registration entries for trials registered on *ClinicalTrials.gov*;
(2) discrepancies between final registry entries and corresponding peer-reviewed publications for trials sourced from *ClinicalTrials.gov*.

Evaluation of changes between the initial and final *ClinicalTrials.gov* entries was performed using the selected TRDS items with the exception of the NCT identifier because this registry-assigned identifier remains constant across registry updates. The denominator for analyses of changes in WHO TRDS items varied because some items were not reported in the initial registration entry or were missing in one of the registry versions. Therefore, changes were assessed only for trials in which the relevant data item was available for comparison. Comparisons between final *ClinicalTrials.gov* registry entries and corresponding publications were conducted using 13 TRDS items. TRDS items 9 and 10 were excluded from these analyses because these items are typically not reported or are modified during journal editorial processing.

Discrepancies in adverse event reporting between registry records and corresponding peer-reviewed publications were assessed for trials with available results data. Data were extracted from the Results section of ClinicalTrials.gov, including the Adverse Events module (serious adverse events and other adverse events), the All-Cause Mortality field, and information reported within outcome results or participant flow. Adverse event reporting was assessed using complementary metrics, including the number of affected participants and the total number of events, to capture both the proportion of patients experiencing adverse events and the overall event frequency.

Comparisons between registry records and publications included the number of patients experiencing adverse events, the total number of reported adverse events, the description and terminology used for adverse events and, where applicable, the frequency threshold used for reporting adverse events in publications.

Discrepancies were categorized as differences in the number of affected participants, the number of reported events, differences in event descriptions, or omission of events in the publication. When publications did not provide sufficient information to determine the number of affected participants or events, the result was classified as unable to determine.

When the Final Rule was implemented, *ClinicalTrials.gov* introduced an ACM field to trial records. As a result, trials completed before this change reported deaths as adverse events or outcomes because there was no specific field to document participant deaths [24]. Therefore, for trials completed before 18 January 2017, we considered participant deaths as reported only if they were explicitly mentioned anywhere in the Results section on *ClinicalTrials.gov*. For trials completed on or after 18 January 2017, we evaluated the reporting of participant deaths both in the ACM field and elsewhere in the Results section on *ClinicalTrials.gov*. If the number of affected patients was not clearly reported as zero or ≥1 in the ACM field or elsewhere in the Results section, ACM was considered not reported. Similarly, SAEs and OAEs were regarded as reported only when explicitly stated as zero or with frequencies of one or more on *ClinicalTrials.gov* and in publications. Trials that did not explicitly list zero in these fields were treated as not reporting zero deaths. For determining discrepancies between the registry and publications, not applicable (N/A) was recorded for trials that only had OAEs, SAEs, or ACM reported in one source, which precluded comparisons.

Two investigators independently extracted data in parallel from the entire trial cohort and their corresponding publications (IJMC and SP) to minimize the risk of subjective interpretation bias. SP and AT assessed the completeness of results reporting for trials registered in *ClinicalTrials.gov* and for their corresponding peer-reviewed publications. Inter-rater reliability was generally high for SAE reporting between ClinicalTrials.gov and publications, with kappa values of 1.00 for most SAE elements. For OAE and ACM reporting, inter-rater reliability was also high, with kappa values ranging from 0.94 to 1.00 for OAEs and from 0.76 to 1.00 for ACM. Lower agreement was observed for the interpretation of the presence or absence of SAEs in publications (kappa 0.17; 95% CI −0.19 to 0.52) and for OAE descriptions between ClinicalTrials.gov and publications (kappa 0.44; 95% CI 0.07 to 0.81). Discordant assessments were resolved through consensus discussion.

### 2.4. Data Analysis

Data extracted from *ClinicalTrials.gov* and corresponding peer-reviewed publications were entered into a spreadsheet and coded for analysis. The data set will be available in Zenodo. Descriptive statistics were used to summarize the data. Data analysis was performed using MedCalc Statistical Software version 17.9.4 (MedCalc Software Ltd., Ostend, Belgium). Categorical variables were reported as frequencies and percentages. Continuous non- parametric variables were summarized using medians with interquartile ranges (IQRs) and ranges, or medians with 95% confidence intervals (CIs), as appropriate. Differences in adverse event reporting between registry records and publications were assessed using categorical variables indicating the presence or absence of reported events. When information could not be categorized based on the available data, it was classified as unable to determine or as no values > 0 reported in publications along with the reason for non-categorization.

## 3. Results

### 3.1. General Characteristics of Transfusion Medicine Clinical Trials from ClinicalTrials.gov

**Figure 1:**
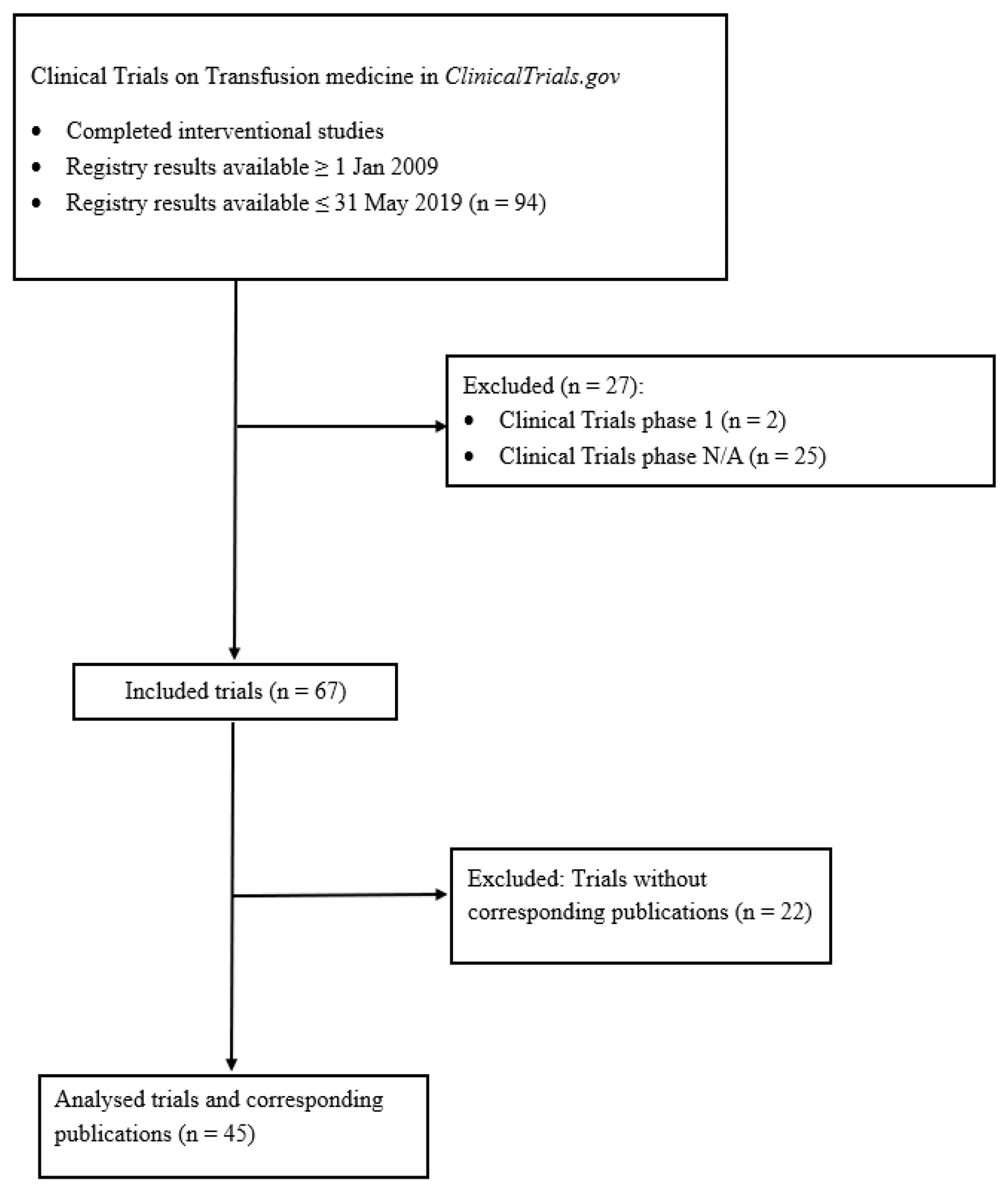
Flow diagram of transfusion medicine clinical trials from *ClinicalTrials.gov*.

Of the 94 clinical trials in transfusion medicine that met the predefined eligibility criteria, 67 trials remained after exclusion of ineligible study phases (Figure 1). Corresponding peer-reviewed journal publications were identified for 45 trials (67%), which were included in comparative analyses.

General characteristics at the time of the final registration update were analyzed for the full cohort of trials registered in *ClinicalTrials.gov* (Table 1). A complete list of all registered trials and their characteristics is provided in the Supplementary Materials. Most trials were randomized, phase 2 or phase 3 studies, with an open-label design and a parallel-group intervention model, and the majority were sponsored by industry or academic institutions.

Trials were further grouped into predefined thematic categories to provide an overview of research priorities within transfusion medicine (Table 2). Patient blood management-related interventions were most frequent (24/67, 36%), followed by iron chelation therapies (15/67, 22%) and studies evaluating blood components (13/67, 19%). Iron chelation studies were retained because they involved transfusion-dependent patient populations and met the predefined eligibility criteria applied across the study cohort. Hemostatic and coagulation interventions accounted for 15% of trials, while immunohematology and transplantation-related studies were least frequently represented.

Median elapsed times across key registration and publication milestones indicate that trials were typically registered at study initiation and required just over three years to reach completion (Table 3). Registry results were generally available approximately one and a half years after primary completion, while journal publications appeared a median of four months earlier than registry postings, with substantial variability across trials.

### 3.2. Completeness of WHO TRDS Reporting at Initial Registration Entry, Final Registration Update, and Corresponding Peer-Reviewed Publications in Transfusion Medicine Clinical Trials from ClinicalTrials.gov

Missing items from the WHO TRDS were evaluated at three time points: the initial registration entry (*n* = 67), the final registration update (*n* = 67), and the corresponding peer-reviewed journal publications (*n* = 45) (Table 4).

At initial registration, missing data were observed for several TRDS items. The most frequently missing item was completion date (22/67, 33%), followed by key secondary outcomes (17/67, 25%), key primary outcomes (10/67, 15%), countries of recruitment (9/67, 13%), and date of first enrolment (9/67, 13%). Missing information was less common for scientific title (4/67, 6%), sample size (4/67, 6%), and key exclusion criteria (2/67, 3%). No missing data were identified for the trial registration number (NCT identifier), primary sponsor, public title, health condition studied, interventions, key inclusion criteria, or study type at initial registration.

By the final registry update, the overall number of missing items had markedly decreased. Missing data persisted primarily for key secondary outcomes (12/67, 18%), while only isolated instances of missing information remained for countries of recruitment (3/67, 4%). All other TRDS items, including the trial registration number (NCT identifier), were complete in the final registration update.

In contrast, peer-reviewed journal publications exhibited substantial missing information for several TRDS items. The most frequently missing items were completion date (29/45, 64%) and date of first enrolment (25/45, 56%). The trial registration number (NCT identifier) was missing in 6 of 45 publications (13%), despite being consistently reported in both initial and final registry entries. Missing data were also observed for the IPD sharing statement (2/8, 25%), primary sponsor (4/45, 9%), and countries of recruitment (4/45, 9%). No missing data were identified for scientific title, health condition studied, interventions, key inclusion criteria, key exclusion criteria, sample size, or key primary and secondary outcomes in journal publications. Public title was not assessed in publications, whereas missing study type information reflected omission of trial phase only.

Among trials with complete WHO TRDS reporting at initial registration (*n* = 22), 5 (23%) were industry-sponsored and 14 (64%) were prospectively registered. At the final registration update, 25 of 52 trials (48%) were industry-sponsored and 28 (54%) were prospectively registered (Table 5).

### 3.3. Changes in WHO TRDS Reporting Between the Initial Registration Entry and Final Registration Update in Transfusion Medicine Clinical Trials from ClinicalTrials.gov

Across the 67 clinical trials, changes in WHO TRDS items between the initial registration entry and the final registration update on *ClinicalTrials.gov* were common, although the extent of change varied substantially across individual items (Table 6).

The most frequently updated items were completion date (40/45, 89%) and key secondary outcomes (41/46, 89%), followed by sample size (55/63, 87%) and key primary outcomes (49/57, 86%). Intervention-related information was also frequently modified (45/67, 67%).

Moderate levels of change were observed for key inclusion criteria (27/67, 40%), date of first enrolment (20/58, 34%), key exclusion criteria (19/65, 29%), and countries of recruitment (14/57, 25%). Descriptive fields such as public title (19/67, 28%), scientific title (14/63, 22%), health condition studied (15/67, 22%), and primary sponsor (11/67, 16%) were less frequently updated, while study type showed no changes.

### 3.4. Changes in WHO TRDS Reporting Between the Final Registration Update and Corresponding Peer-Reviewed Journal Publications in Transfusion Medicine Clinical Trials from ClinicalTrials.gov

Changes between the final registration update and corresponding peer-reviewed journal publications were common across multiple WHO TRDS items (Table 7). The most frequent discrepancies were observed for key exclusion criteria (35/45, 78%) and key inclusion criteria (31/45, 69%). Substantial discrepancies were also identified for key secondary outcomes (30/45, 67%) and study type (22/45, 49%), as well as for sample size (17/45, 38%).

Changes in primary sponsor were identified in 18 of 41 trials (44%); in one-third of these cases (6/18, 33%), the publication reverted to reporting the original sponsor listed at initial registration. Differences in recruitment countries were observed in 6 of 41 trials (15%).

Temporal inconsistencies were also frequent. Changes in the date of first enrolment occurred in 10 of 20 trials (50%), and discrepancies in the completion date were present in 8 of 16 trials (50%). In contrast, discrepancies were less frequent for key primary outcomes (8/45, 18%) and uncommon for health condition studied and interventions (each 1/45, 2%). No discrepancies were observed for the trial registration number (NCT identifier).

### 3.5. Adverse Event Reporting in Transfusion Medicine Clinical Trials from ClinicalTrials.gov and Corresponding Publications

AE reporting in transfusion medicine trials (Table 8) showed that, among the 45 trials with available AE data, SAEs were reported in 31 trials (69%) in ClinicalTrials.gov and in 26 publications (58%), while OAEs were reported in 32 trials (71%) in *ClinicalTrials.gov* and in 29 publications (64%). In addition, SAEs and OAEs were not separately or explicitly reported in 1 trial (2%) in the registry and in 9 publications (20%).

Deaths were reported in 20 trials (44%) in *ClinicalTrials.gov* and in 27 publications (60%). Within the registry, deaths were reported across different sections, including 8 trials (40%) in the ACM field, 8 trials (40%) in outcome results or participant flow, and 4 trials (20%) within the adverse event module.

A subset of trials explicitly reported zero adverse events. Zero SAEs were reported in 12 trials (27%) in the registry and in 4 publications (9%), while zero OAEs were reported in 11 trials (24%) in the registry and in 5 publications (11%). Zero deaths were reported in 6 trials (13%) in the registry and in 3 publications (7%), whereas no deaths were reported in 19 registry entries (42%) and 15 publications (33%).

The number of patients experiencing adverse events per trial varied widely. For SAEs, the median number of affected patients per trial was 17 in the registry and 16 in publications, with ranges of 0–365 and 0–1025, respectively. For OAEs, the median number of affected patients per trial was 33 in the registry and 40 in publications, with ranges extending up to 607 patients per trial in both sources.

### 3.6. Discrepancies in Serious Adverse Event Reporting for Transfusion Medicine Clinical Trials from ClinicalTrials.gov and Corresponding Publications

Discrepancies in the reporting of SAEs were common (Table 9). Differences in the number of patients with SAEs were identified in 21/45 trials (47%), whereas 13 (29%) showed no discrepancies. In the remaining 11 (24%) trials, assessment was not possible due to the absence of reported values >0 in publications or because adverse events were not clearly distinguishable.

Among trials with discrepancies (*n* = 21), more patients were reported in 12 registry trials (57%), whereas publications reported higher numbers in 9 trials (43%). Differences in the total number of reported SAEs between sources were observed in 24/45 trials (53%), most often with higher numbers in the registry (19/24, 79%) than in publications (5/24, 21%).

Discrepancies in the description of SAEs were identified in 21 trials (47%), while 8 (18%) showed consistent reporting. In the remaining 16 (35%) trials, comparison was not possible due to insufficient or non-differentiated reporting of adverse events. Additionally, omission of one or more registered SAEs in publications was observed in 14 (31%) trials, whereas 22 (49%) showed no such omissions, and in 9 (20%) trials assessment was not possible.

### 3.7. Discrepancies in Other Adverse Event Reporting for Transfusion Medicine Clinical Trials from ClinicalTrials.gov and Corresponding Publications

Discrepancies in OAE reporting were frequent (Table 10). Differences in the number of patients with OAEs were identified in 13/45 trials (29%), whereas 9 (20%) showed no discrepancies. In the remaining 23 (51%) trials, assessment was not possible due to the absence of reported values >0 in publications or because adverse events were not clearly distinguishable.

Among trials with discrepancies (*n* = 13), higher patient counts were reported in 4 registry trials (31%), whereas publications reported higher numbers in 9 trials (69%). Differences in the total number of reported OAEs between sources were observed in 14/45 trials (31%), with no consistent predominance of either source; higher numbers were reported in the registry in 8 trials (57%) and in publications in 6 trials (43%).

Discrepancies in the description of OAEs were identified in 14 trials (31%), while 7 (16%) showed consistent reporting. In 24 (53%) trials, comparison was not possible due to insufficient or non-differentiated reporting of adverse events. Additionally, omission of one or more registered OAEs in publications was observed in 18 (40%) trials, whereas 15 (33%) showed no such omissions, and in 12 (27%) trials assessment was not possible.

Regarding reporting characteristics, most trials reported OAEs as adverse events with quantifiable values in 30 trials (67%), followed by reporting without quantifiable values in 10 trials (22%), treatment-emergent adverse event (TEAE)-only reporting in 4 trials (9%), and adverse drug reaction (ADR)-only reporting in 1 trial (2%). Reporting thresholds were rarely consistent between sources, with most publications not stating a threshold in 40 trials (89%). Among trials with omission of registered OAEs (*n* = 18), threshold reporting was present in 4 (22%) trials, and TEAE reporting in 6 (33%) trials.

## 4. Discussion

This study demonstrated that clinical trials in transfusion medicine frequently show inconsistencies in the reporting of key WHO TRDS items and safety outcomes across different stages of the trial life cycle. Although the completeness of registry records improved substantially between initial registration and the final registration update, this improvement was not consistently reflected in corresponding peer-reviewed journal publications [37,38]. Persistent differences between registries and journal articles remain a recognized challenge in ensuring transparency and completeness of clinical trial reporting across medical specialties [39].

When examining earlier stages of the trial life cycle, missing WHO TRDS items at initial registration were predominantly related to study timelines and outcome reporting fields [36]. Similar deficiencies during early registration stages have been described across clinical research despite long-standing requirements for prospective trial registration as a condition for publication [40]. Comparative analyses of registered protocols, trial registries, and published reports demonstrate that discrepancies may emerge at multiple stages of the trial life cycle and across diverse therapeutic fields [41]. In the present cohort, missing information in publications was particularly frequent for dates of first enrolment and trial completion, whereas discrepancies were common for eligibility criteria and outcome specifications. Such omissions may limit the interpretability, generalizability, and reproducibility of published findings, particularly when outcome definitions or timing are affected [42].

Discrepancies between final registry entries and journal publications extended beyond administrative details to key methodological characteristics, including eligibility criteria, study design descriptors, and outcome reporting. While some differences may reflect legitimate protocol amendments or updates during trial conduct, not all identified differences necessarily indicate inadequate reporting. In contrast, changes affecting key methodological elements, such as primary outcomes or sample size, may warrant closer scrutiny, as they could be associated with selective reporting. However, in the absence of transparent documentation of such changes in publications, it remains difficult to distinguish justified updates from potential reporting bias. Accordingly, the observed differences should be interpreted in the context of their potential origin and timing.

Extensive empirical evidence has demonstrated selective outcome reporting and related reporting biases in randomized trials [43,44,45]. Evidence from multiple clinical domains further indicates that discordance between registry records and corresponding publications remains common for essential methodological items, suggesting that this misalignment is not confined to a single specialty or intervention type [46].

Beyond methodological characteristics, inconsistencies were also observed in safety-related reporting elements. Although the overall reporting frequency of serious adverse events was broadly comparable between registry records and corresponding publications, registry entries frequently contained higher numbers and more detailed reporting of serious adverse events within the sources analyzed, whereas publications more often omitted or simplified these data. Previous systematic evaluations have demonstrated substantial variability in the completeness, structure, and detail of adverse event reporting across randomized trials [47,48]. These recognized limitations have directly informed the development of dedicated reporting guidance, including the CONSORT harms extension and the PRISMA harms checklist, which aim to improve transparency and completeness of harms reporting [49,50]. In addition, a considerable proportion of trials could not be directly compared due to merged or non-differentiated reporting of adverse events (e.g., combined SAE/OAE or OAE/TEAE reporting) or the absence of reported values > 0, further limiting interpretability. Inconsistent reporting formats and thresholds contributed to heterogeneity across sources. Journal publications often report adverse events above predefined frequency thresholds, which may contribute to differences in the level of detail compared with registry entries. Furthermore, deaths were reported inconsistently across different registry sections and publications, complicating their identification and comparison. This may partly explain why deaths appeared more frequently in publications despite more extensive serious adverse event reporting within registry records. Such limitations are particularly relevant in transfusion medicine, where interventions are often administered to vulnerable patient populations and accurate harms reporting plays a critical role in balanced benefit–risk assessment [51]. Nevertheless, discrepancies between registry entries and publications should primarily be interpreted in the context of reporting transparency and consistency between evidence sources rather than as a direct indicator of deficiencies in regulatory safety oversight.

These inconsistencies may compromise the accurate interpretation and integration of clinical evidence into patient care and clinical decision-making [52]. When registries and publications diverge, systematic reviewers and guideline developers may face uncertainty regarding which source should be considered authoritative, potentially affecting the completeness and reliability of evidence synthesis [53]. At a broader level, empirical studies highlight persistent infrastructural and regulatory barriers to complete and timely dissemination of trial results in registries, contributing to research waste and inefficiencies in evidence-based medicine [54], while the present findings suggest that inconsistencies and selective reporting may additionally contribute to differences between evidence sources.

Several limitations of our study should be acknowledged. This analysis was restricted to trials registered in *ClinicalTrials.gov*, and retrospective assessment of registries and corresponding publications may introduce some degree of subjectivity in classifying discrepancies. However, the use of predefined coding rules and independent data extraction by experienced reviewers reduces the risk of systematic misclassification, consistent with empirical evidence demonstrating discrepancies between registered and published trial information [55]. In addition, focusing on a single registry may limit generalizability to trials registered exclusively in other primary registries [56]. Some TRDS items introduced or modified in later WHO TRDS versions may not have been applicable to trials registered earlier in the study period. Furthermore, the findings should be interpreted in light of the search strategy applied, which may influence the representativeness of the study sample. This approach was selected to maximize sensitivity within ClinicalTrials.gov; however, reliance on a single keyword and a single registry may result in both over-inclusion (e.g., studies in transfusion-dependent populations) and under-inclusion of relevant trials described using alternative terminology. Restricting the analysis to trials with available registry results may also have preferentially selected a more administratively compliant and publication-active subset of studies. In addition, only the first full publication reporting the main trial results was included in comparative analyses. Because adverse event data may also be reported in secondary or follow-up publications, this approach may have underestimated the extent of safety reporting in journal publications.

Finally, the relatively small sample size may limit the generalizability of the findings, and the study did not assess the potential clinical impact of the identified discrepancies.

Overall, these findings highlight a persistent gap between regulatory reporting expectations and the information presented in peer-reviewed journal articles [37,57]. Despite the availability of structured reporting frameworks and guidance, including WHO TRDS requirements and CONSORT recommendations for harms reporting, achieving concordance between registry records and publications remains challenging in practice, as demonstrated by empirical analyses of trial result reporting and publication timelines [58]. These discrepancies may also reflect the distributed nature of responsibilities in clinical trial reporting, where regulatory safety reporting, trial conduct, and journal publication processes are overseen by different stakeholders without a formally assigned mechanism for routine verification of concordance between registry records and corresponding publications. Journal editors and peer reviewers may contribute to improving reporting consistency by encouraging verification of submitted manuscripts against trial registry records. Greater alignment between registries and publications, particularly for adverse event reporting and safety data transparency, could be supported through consistent application of reporting guidelines and the use of cross-checking procedures during the editorial process [49,50,59].

Further stratified analyses, such as according to trial phase, sponsor type, or intervention category, may provide additional insight into patterns of reporting discrepancies and should be considered in future research.

## 5. Conclusions

This study showed that clinical trials in transfusion medicine frequently exhibit inconsistencies in reporting key trial descriptors and safety outcomes between *ClinicalTrials.gov* records and corresponding peer-reviewed journal publications. Although the completeness of registry information improves throughout the trial registration process, these improvements are not consistently reflected in the published literature, with registry entries often containing more detailed safety reporting within the analyzed sources. Differences affecting methodological details, including eligibility criteria, study timelines, outcomes, and safety reporting, may affect transparency, reproducibility, and the reliability of evidence synthesis. In addition, substantial heterogeneity in adverse event reporting, including inconsistent terminology, reporting formats, and thresholds, limits direct comparability across trials.

These findings emphasize the need for closer alignment between trial registries and publications across the clinical trial life cycle. Improving the visibility and traceability of protocol changes, alongside more structured approaches to results reporting, may help strengthen confidence in trial evidence and support more reliable translation of research findings into clinical practice in transfusion medicine.

## Figures and Tables

**Table 1:** General characteristics of transfusion medicine clinical trials from *ClinicalTrials.gov*.

Baseline Characteristics	No. of Trials (%) ^a^
**Trial phase:**	
1/2	5 (7)
2	28 (42)
2/3	4 (6)
3	20 (30)
4	10 (15)
**Masking** ^b^:	
None (Open-label)	34 (51)
Single-blind	9 (13)
Double-blind	7 (10)
Triple-blind	7 (10)
Quadruple-blind	10 (15)
**Intervention model:**	
Parallel assignment	41 (61)
Single assignment	22 (33)
Crossover assignment	4 (6)
**Primary sponsor** ^c^:	
National Institutes of Health	2 (3)
Industry	27 (40)
Community-based organization	9 (13)
University	29 (43)
**Type of intervention:**	
Drug	39 (58)
Drug & Procedure	5 (7)
Drug & Biological	1 (1)
Drug & Radiation	1 (1)
Drug & Other investigational products	3 (4)
Procedure	5 (7)
Procedure & Biological	1 (1)
Biological	9 (13)
Biological & Other investigational products	1 (1)
Biological & Device	1 (1)
Other investigational products	1 (1)
**Allocation** ^d^:	
Nonrandomized	10 (15)
Randomized	46 (69)
Not applicable	11 (16)
**Primary purpose:**	
Treatment	56 (84)
Prevention	7 (10)
Supportive care	1 (1)
Basic science	1 (1)
Other	1 (1)
Missing	1 (1)

^a^ Percentages were calculated using the total number of included trials as the denominator (*n* = 67) and may not add to 100% due to rounding. ^b^ The categories were defined according to *ClinicalTrials.gov* and entered into the registry by the sponsor. No attempt was made to verify the definitions of masking types. ^c^ The categorization was based on *ClinicalTrials.gov* registry entries. ^d^ The ‘Not applicable’ category refers to studies in which randomization or an allocation model was not applicable.

**Table 2:** Distribution of transfusion medicine clinical trials from *ClinicalTrials.gov* by main categories.

Main Categories ^a^	No. of Trials (%) ^b^
Blood components	13 (19)
Hemostasis & Coagulation	10 (15)
Immunohematology	3 (4)
Patient Blood Management (PBM) ^c^	24 (36)
Chelation & Iron overload	15 (22)
Transplantation	2 (3)

^a^ Trials were assigned to a single main category based on the dominant intervention reported in *ClinicalTrials.gov*, even if multiple transfusion-related components were involved. ^b^ Percentages were calculated using the total number of included trials as the denominator (*n* = 67) and may not add to 100% due to rounding. ^c^ The Patient Blood Management (PBM) category includes both pharmacological and non-pharmacological strategies aimed at reducing transfusion exposure (e.g., transfusion thresholds, erythropoiesis-stimulating agents, immunomodulatory agents and resuscitation strategies).

**Table 3:** Time intervals (months) between key trial milestones for transfusion medicine clinical trials from *ClinicalTrials.gov*.

Different Time Points for the Included Clinical Trials	Median Months (95% CI)
Initial registration entry to Study start date ^a^	0.00 (0.00 to 0.00)
Study start date to Study completion date ^a^	38.00 (33.00 to 49.71)
Primary completion date to Results posting date ^a^	19.77 (15.55 to 25.87)
Study completion date to Study publication date ^b^	12.43 (8.97 to 21.18)
Results posting date to Study publication date ^b^	−3.77 (−16.52 to 3.50)

^a^ Calculated for all trials with available registry data (*n* = 67). ^b^ Calculated for trials with both posted results and a corresponding peer-reviewed publication (*n* = 45).

**Table 4:** Missing WHO Trial Registration Data Set (TRDS) items at initial registration, final registration update, and in corresponding peer-reviewed journal publications for transfusion medicine clinical trials.

WHO TRDS Items Missing	Initial Registration Entry (*n* = 67)	Final Registration Update (*n* = 67)	Journal Publication (*n* = 45)
NCT identifier	0 (0)	0 (0)	6 (13)
Primary sponsor	0 (0)	0 (0)	4 (9)
Public title ^a^	0 (0)	0 (0)	–
Scientific title	4 (6)	0 (0)	0 (0)
Countries of recruitment	9 (13)	3 (4)	4 (9)
Health condition studied	0 (0)	0 (0)	0 (0)
Interventions	0 (0)	0 (0)	0 (0)
Key inclusion criteria	0 (0)	0 (0)	0 (0)
Key exclusion criteria	2 (3)	0 (0)	0 (0)
Study type ^b^	0 (0)	0 (0)	18 (40)
Date of first enrolment	9 (13) ^c^	0 (0)	25 (56) ^d^
Sample size	4 (6)	0 (0)	0 (0)
Key primary outcomes	10 (15)	0 (0)	0 (0)
Key secondary outcomes	17 (25)	12 (18)	0 (0)
Completion date	22 (33) ^e^	0 (0)	29 (64) ^f^
IPD sharing statement ^g^	–	–	2 (25)

^a^ Public title item was not analyzed for publications due to their routine exclusion from the journal articles. ^b^ Discrepancies reflected omission of trial phase information. ^c^ A partially reported date was identified in 4 trials. ^d^ A partially reported date was identified in 3 trials. ^e^ A partially reported date was identified in 1 trial. ^f^ A partially reported date was identified in 2 trials. ^g^ IPD sharing statement refers to a declaration on the intended sharing of deidentified individual participant-level data. Under the WHO TRDS, this item became mandatory for journal articles published after 1 July 2018. It was applicable to 8 publications in this study; therefore, percentages were calculated using n = 8 as the denominator. No trials met criteria for IPD completeness, as all were initiated before the introduction of this item on 6 November 2017.

**Table 5:** Funding source and registration timing among trials with complete WHO TRDS reporting in *ClinicalTrials.gov*.

Registration Stage	Trials with Complete WHO TRDS ^a^, *n*	Industry-Sponsored, *n* (%)	Prospectively Registered, *n* (%)
Initial registration	22	5 (23)	14 (64)
Final registration update	52	25 (48)	28 (54)

^a^ Complete WHO TRDS reporting was defined as the presence of all evaluated WHO TRDS items at the specified registration stage.

**Table 6:** Changes in WHO Trial Registration Data Set (TRDS) items between initial registration entry and final registration update for transfusion medicine clinical trials.

WHO TRDS Items Changed	Initial Registration Entry to Final Registration Update (Maximum *n* = 67), no. (%) ^a^
Primary sponsor	11/67 (16)
Public title	19/67 (28)
Scientific title	14/63 (22)
Countries of recruitment	14/57 (25)
Health condition studied	15/67 (22)
Interventions	45/67 (67)
Key inclusion criteria	27/67 (40)
Key exclusion criteria	19/65 (29)
Study type	0 (0)
Date of first enrolment	20/58 (34)
Sample size	55/63 (87)
Key primary outcomes	49/57 (86)
Key secondary outcomes	41/46 (89)
Completion date	40/45 (89)
IPD sharing statement	–

^a^ Denominators vary across WHO TRDS items because changes were assessed only among trials in which the corresponding item was reported at both the initial registration entry and the final registration update. The IPD sharing statement was not assessed, as this item was introduced after initiation of all included trials.

**Table 7:** Changes in WHO Trial Registration Data Set (TRDS) items between the final registration update and corresponding peer-reviewed journal publications for transfusion medicine clinical trials.

WHO TRDS Items Changed	Final Registration Entry to Publication (Maximum *n* = 45), No. (%)
NCT number	0/39 (0)
Primary sponsor ^a^	18/41 (44)
Countries of recruitment	6/41 (15)
Health condition studied	1/45 (2)
Interventions	1/45 (2)
Key inclusion criteria	31/45 (69)
Key exclusion criteria	35/45 (78)
Study type ^b^	22/45 (49)
Date of first enrolment ^c^	10/20 (50)
Sample size	17/45 (38)
Key primary outcomes ^d^	8/45 (18)
Key secondary outcomes ^e^	30/45 (67)
Completion date ^f^	8/16 (50)

^a^ 6/18 discrepant cases reflected reporting of the original sponsor rather than the sponsor listed in the final registry entry. ^b^ Discrepancies included missing phase information in the publication (18/45), changes in study design (3/45), and other changes such as the addition of a non-inferiority design in the publication (1/45). In one publication, two distinct changes were identified and coded separately. ^c^ Discrepancies included later dates reported in the publication (6/20) and earlier dates reported in the publication (4/20). ^d^ Discrepancies included newly introduced outcomes (1/45), omission of registered outcomes (2/45), switching between primary and secondary outcomes (4/45), and differences in outcome time frames (1/45). ^e^ Discrepancies included newly introduced outcomes (15/45), omission of registered outcomes (13/45), newly introduced outcomes reported as secondary (1/45), and combinations of newly introduced and omitted outcomes (1/45). ^f^ Discrepancies included later dates reported in the publication (3/16), earlier dates reported in the publication (5/16).

**Table 8:** Adverse event reporting in transfusion medicine clinical trials and corresponding peer-reviewed journal publications.

	*ClinicalTrials.gov*, No. (%)	Publications, No. (%)
**AEs > 0 reported (*n* = 45) ^a^**		
SAEs	31 (69)	26 (58)
OAEs	32 (71)	29 (64)
SAEs and OAEs not separately reported or not explicitly reported	1 (2) ^b^	9 (20) ^c^
Deaths	20 (44)	27 (60)
**Deaths reported in *ClinicalTrials.gov* (*n* = 20)**		
In the All-Cause mortality field	8 (40)	–
In outcome results or participant flow	8 (40)	–
In the adverse event module	4 (20)	–
**AEs reported as zero (*n* = 45)**		
SAEs	12 (27)	4 (9)
OAEs	11 (24)	5 (11)
Deaths	6 (13)	3 (7)
No deaths reported	19 (42)	15 (33)
**Number of patients with AEs per trial (median, IQR/range) ^d,e^**		
SAEs	17, 0–72/0–365	16, 3–66/0–1025
OAEs	33, 0–130/0–607	40, 0–168/0–607

Abbreviations: AE, adverse event; SAE, serious adverse event; OAE, other adverse event. ^a^ Within each source (*ClinicalTrials.gov* and publications), reporting categories for SAEs, OAEs, and deaths sum to the total number of trials (*n* = 45). Categories are not mutually exclusive. Cross-source reporting patterns (e.g., events reported in only one source) were not presented as separate categories but are reflected within the overall reporting groups. ^b^ NCT00529152—adverse events were reported only within an outcome without separate classification of SAEs and OAEs. ^c^ Publications in which SAEs and OAEs were not separately distinguishable: NCT00529152, NCT00838331, NCT01178281, NCT01227005, NCT01370406, NCT01545232, NCT01611935, NCT01651806, and NCT01736683. ^d^ Deaths were not consistently reported as directly comparable frequencies across trials; therefore, only source-level reporting patterns were assessed. ^e^ NCT00529152 was excluded from the calculation of median SAEs because adverse events were reported only within an outcome.

**Table 9:** Discrepancies in reported serious adverse events in transfusion medicine clinical trials and corresponding peer-reviewed journal publications.

Number of Patients with SAEs (*n* = 45)	No. (%)
Yes	21 (47)
No	13 (29)
Unable to determine or not applicable ^a^	11 (24)
**Among trials with differences (*n* = 21)**	
More in the registry	12 (57)
More in the publication	9 (43)
**Number of SAEs that differ between sources (*n* = 45)**	
Yes	24 (53)
No	11 (24)
Unable to determine or not applicable ^b^	10 (22)
**Among trials with differences (*n* = 24)**	
More in the registry	19 (79)
More in the publication	5 (21)
**Different description of SAEs (*n* = 45)**	
Yes	21 (47)
No	8 (18)
Unable to determine or not applicable ^c^	16 (35)
**Omission of 1 or more registered SAEs in publications (*n* = 45)**	
Yes	14 (31)
No	22 (49)
Unable to determine or not applicable ^d^	9 (20)

Abbreviations: AE, adverse event; SAE, serious adverse event; OAE, other adverse event. ^a^ Unable to determine due to merged and indistinguishable adverse event reporting (*n* = 6) or absence of values > 0 in publications (*n* = 5). ^b^ Unable to determine due to uninterpretable values (*n* = 5) or absence of values >0 in publications (*n* = 5). ^c^ Unable to determine due to merging of SAEs and OAEs (n = 5) or absence of values >0 in publications (*n* = 11). ^d^ Unable to determine due to merging of OAEs and SAEs (n = 1) or absence of reporting (not applicable) (*n* = 8).

**Table 10:** Discrepancies in reported other adverse events in transfusion medicine clinical trials and corresponding peer-reviewed journal publications.

Number of Patients with OAEs (*n* = 45)	No. (%)
Yes	13 (29)
No	9 (20)
Unable to determine or not applicable ^a^	23 (51)
**Among trials with differences (*n* = 13)**	
More in the registry	4 (31)
More in the publication	9 (69)
**Number of OAEs that differ between sources (*n* = 45)**	
Yes	14 (31)
No	9 (20)
Unable to determine or not applicable ^b^	22 (49)
**Among trials with differences (*n* = 14)**	
More in the registry	8 (57)
More in the publication	6 (43)
**Different description of OAEs (*n* = 45)**	
Yes	14 (31)
No	7 (16)
Unable to determine or not applicable ^c^	24 (53)
**Type of reporting (*n* = 45)**	
Reported as an AE with quantifiable values	30 (67)
Reported as an AE without quantifiable values	10 (22)
Reported as TEAE only	4 (9)
Reported as ADR only	1 (2)
**Frequency threshold (*n* = 45)**	
Same in both sources	2 (4)
Higher in registry	1 (2)
Higher in publication	2 (4)
Unstated in publications	40 (89)
**Omission of 1 or more registered OAEs in publications (*n* = 45)**	
Yes	18 (40)
No	15 (33)
Unable to determine or not applicable ^d^	12 (27)
**Among trials with omission (*n* = 18)**	
**Threshold reporting**	
Yes	4 (22)
No	14 (77)
**TEAE-only reporting**	
Yes	6 (33)
No	12 (66)

Abbreviations: AE, adverse event; SAE, serious adverse event; OAE, other adverse event; TEAE, treatment-emergent adverse event, ADR, adverse drug reaction ^a^ Unable to determine due to combination reporting of the most common OAEs and TEAEs in the publication (*n* = 12) and no values > 0 reported in publications (*n* = 11). ^b^ Unable to determine due to merged OAE and SAE reporting (*n* = 10) and no values > 0 reported in publications (not applicable) (*n* = 12). ^c^ Unable to determine due to merged OAE and SAE reporting (*n* = 4) and no values >0 reported in publications (not applicable) (*n* = 20). ^d^ Unable to determine due to merged OAE and SAE reporting.

## Data Availability

The data set generated during the current study is available in the Supplementary Materials. It includes all extracted variables and the coding framework applied in the comparative analyses necessary to reproduce the results reported in this article.

## Supplementary Materials

The following supporting information can be downloaded at: https://www.mdpi.com/article/10.3390/jcm15103981/s1.
