# Supplementary material for: Consistency Between Clinical Trial Registry Entries and Journal Publications in Transfusion Medicine: An Observational Study
Source: J Clin Med. 2026 May 21;15(10):3981. doi: 10.3390/jcm15103981 (PMC13207823; doi:10.3390/jcm15103981)
Supplement: Supplementary file 1 [file jcm-15-03981-s001.zip › Table S6. Database coding keys.pdf]

Trial general characteristics		
Field name		Definition
1. Brief (WHO: Public) title brieftitl_init (column N) brieftitl_last (column O)<br>2. Official (WHO: Scientific) title officialtitl_init (column P) officialtitl_last (column Q)		0 = No; 1 = Yes
3. Masking masking_init (column S) masking_last (column T)		0= none (open label); 1= single blind masking; 2= double blind masking; 3 = masked roles unspecified/missing, 4= quadriple 5=triple
4. Allocation allocation_init (column U) allocation_last (column V)		0 = Non-randomized; 1 = Randomized; 2 = N/A
5. Interventional model interventional_model_init (column W) interventional_model_last (column X)		0 = Single Group<br>1 = Parallel<br>2 = Cross-over<br>3 = Factorial<br>4 = Inaccurate entry<br>5 = Not applicable (N/A)<br>6 = Unspecified
6. Primary purpose purpose_init (column Y) purpose_last (column Z)		0 = Treatment<br>1 = Prevention<br>2 = Diagnostic<br>3 = Supportive Care<br>4 = Screening<br>5 = Health Services Research<br>6 = Basic Science<br>7 = Other<br>8 = Inaccurate entry<br>9 = Missing<br>10 = Not applicable (N/A)
7. Phase phase_init (column AA) phase_last (column AB)		0=**N/A**: for trials without phases (e.g., trials of devices or behavioral interventions)<br>1=**Phase 0**: exploratory trials, involving very limited human exposure, with no therapeutic or diagnostic intent (e.g., screening studies, microdose studies).<br>2=**Phase 1**: includes initial studies to determine the metabolism and pharmacologic actions of drugs in humans, the side effects associated with increasing doses, and to gain early evidence of effectiveness; may include healthy participants and/or patients<br>3=**Phase 1/Phase 2**: for trials that are a combination of phases 1 and 2

		4=**Phase 2**: includes controlled clinical studies conducted to evaluate the effectiveness of the<br>drug for a particular indication or indications in patients with the disease or condition<br>under study and to determine the common short-term side effects and risks<br>5=**Phase 2/Phase 3**: for trials that are a combination of phases 2 and 3<br>6=**Phase 3**: includes expanded controlled and uncontrolled trials after preliminary evidence<br>suggesting effectiveness of the drug has been obtained, and are intended to gather<br>additional information to evaluate the overall benefit-risk relationship of the drug and<br>provide an adequate basis for physician labeling<br>7=**Phase 4**: studies of FDA-approved drugs to delineate additional information including the<br>drug's risks, benefits, and optimal use
8. Primary outcome measure<br>prim_outcome_init (column AC)<br>prim_outcome_last (column AD)		0 = no; 1 = yes
9. Secondary outcome measure<br>sec_outcome_init (column AE)<br>sec_outcome_last (column AF)		0 = no; 1 = yes
10. Intervention type<br>inter_type_init (column AG)<br>inter_type_last (column AH)		0=Drug (including placebo)<br>1=Device (including sham)<br>2=Procedure/surgery<br>3=Biological/vaccine<br>4=Behavioral (e.g., psychotherapy, lifestyle counseling)<br>5=Genetic (including gene transfer, stem cell and recombinant DNA)<br>6=Dietary supplement (e.g., vitamins, minerals)<br>7=Radiation<br>8=Other investigational or readily available products
11. Sponsor<br>sponsor_init (column AI)<br>sponsor_last (column AJ)		0= NIH, 1= other U.S. Federal Agency, 2= industry, 3= individual, 4= university, 5= community-based organization

Last registration to publication completeness and changes		
1. NCT number<br>uniq_num (column B)		0=same as in last registration as in article<br>1=changed in article<br>2=not listed in article
2. Primary sponsor<br>prim_sponsor (column C)		0= same as in last registration as in article<br>1= changed in article<br>2= not listed in article<br>3= added at last change before publication<br>4= changed from original to current sponsor in register, but again original in publication<br>5=changed to original in register, but different in publication
3. Countries of recruitment<br>country (column D)		0= same as in last registration as in article<br>1= changed in article from that listed in registry<br>2= not listed in article but in registry<br>4= same in register, but additional ones added in publication<br>5= same in register, but some missing in publication<br>6= not listed in registry but in article
4. Health condition studied<br>cond_pap (column E)		0= same in last registration as in article<br>1= changed in article<br>2= missing in registry<br>3= missing in publication
5. Intervention<br>int_pap (column F)		0=same in last registration as in article<br>1= changed in article<br>2= missing in registry<br>3= missing in publication
6. Key inclusion criteria<br>incl_pap (column G)		0= new criteria added in article<br>1= criteria/on omitted in article<br>2= missing in registry<br>3= same
7. Key exclusion criteria<br>excl_pap (column H)		0= new criteria added in article<br>1= criteria/on omitted in article<br>2= missing in registry<br>3= same
8. Study type<br>study typ_pap (column I)		0= same<br>2= study design change<br>3= phase change<br>4= other changes<br>6= phase missing in publication
9. Date of first enrolment<br>first _enrolment_date (column J)		0= changed to later date (according to date in article vs. in registry)<br>1= changed to earlier date (according to date in article vs. in registry)<br>3= missing in publication<br>4= same<br>5= missing in registry
10. Completion date		0= changed to later date (according to date in article vs. in

completion_date (column K)		registry)<br>1= changed to earlier date (according to date in article vs. in registry)<br>3= missing in publication<br>4= same<br>5= missing in registry
11. Sample<br>sample size_pap_ (column L)		0= greater in article than in registry<br>1= smaller in article than in registry<br>2= missing in registry<br>3= missing in publication<br>4= same
12. Key primary outcomes<br>primary_outcome_pap (column M)		0= new outcomes introduced in publication<br>1= registered outcomes omitted in publication<br>2= outcomes switched, i.e., primary changed to secondary outcome in publication or vice versa<br>3= newly introduced and reported as secondary<br>4= other changes<br>7= combo of newly introduced or omitted outcomes in publication<br>9= same
13. Key secondary outcomes<br>secondary_outcome_pap_ (column N)		0= new outcomes introduced in publication<br>1= registered outcomes omitted in publication<br>2= outcomes switched, i.e., primary changed to secondary outcome in publication or vice versa<br>3= newly introduced and reported as secondary<br>4= other changes<br>7= combo of newly introduced or omitted outcomes in publication<br>9= same

Registry results and publication comparisons		
1. Results: Participant flow (Recruitment details, pre-assignment details, reporting groups		0 = omitted, 1 = not omitted<br><br>These data are not reported in publications in this way.
2. Results: Participant flow (Overall Participant Flow in registry & paper)		0 = omitted, 1 = not omitted<br>If not: same as in publication 0 = no, 1 = yes
3. Results: Baseline characteristics (Population description in registry & paper), Baseline Measures) sample_size, sample_age, sample_gender, sample_region		0 = omitted, 1 = not omitted<br><br>If not: same as in publication 0 = no, 1 = yes;
4. Results: Outcome measures (Participant analysis inclusion type in the registry and paper for primary and secondary outcome)		1 = ITT (Intention-to-treat)<br>2 = per protocol<br>3 = other<br>4 = omitted
5. Results: Outcome measures (Primary and secondary outcome measured values in registry and paper, respectively)		0 = no, descriptive statistics not used, 1 = yes descriptive statistics used in the registry or paper; 2 = no inferential statistics used, 3 = yes inferential statistics used in the registry or paper; 4 = both descriptive and inferential statistics used in the registry or paper;
6. Results: Outcome measures descriptive statistics used (Primary and secondary outcome measured values in registry and paper, respectively)		If outcome measured values have inferential statistics (P-value or 95% CI generated) used then compared to paper (type P-value or 95% CI): 1 = favors experimental; 2 = favors control; 3 = cannot be determined
7. Results: Serious adverse events (SAEs)		SAEs present in registry: 0 = no, 1 = yes for registry & paper, respectively<br>Omitted in publication: 0 = no, 1 = yes<br>Reported as zero or not occurring: 0 = no, 1 = yes

		Different absolute number and/or frequencies 0 = no, 1 = yes<br>If yes: less in register-1, more in register-2;<br>Description of SAE: different from publication 0 = no, 1 = yes
8. Results: Other Adverse events (OAEs)		0 = omitted frequency threshold, 1 = not omitted (type specific %) for registry & paper<br>AEs present in registry: 0 = no, 1 = yes<br>Omitted in publication: 0 = no, 1 = yes<br>Reported as zero or not occurring: 0 = no, 1 = yes<br>Different absolute number and/or frequencies no-1 yes-2<br>If yes: less in register-1, more in register-2;<br>Description of OAE: different from publication 0 = no, 1 = yes
9. Deaths (All-Cause Mortality in the registry)		0 = no, deaths recorded in registry, 1 = yes deaths recorded in registry (registry & paper, respectively)<br>Omitted in publication 0 = no, 1 = yes<br>Reported as zero or not occurring 0 = no, 1 = yes<br>Different absolute number and/or frequencies 0 = no, 1 = yes<br>If yes; less in register-1, more in register-2<br>Description of Deaths: different from publication 0 = no, 1 = yes
10. Participants' discontinuation due to AE		0 = omitted in registry ; 1 = not omitted<br>If not omitted: same as in publication 0 = no, 1 = yes; 3 = not listed<br>If not omitted: less in register-1, more in register-2;
